# Advancing the Science and Management of Renal Cell Carcinoma: Bridging the Divide between Academic and Community Practices

**DOI:** 10.3390/jcm9051508

**Published:** 2020-05-17

**Authors:** Nicholas J. Salgia, Errol J. Philip, Mohammadbagher Ziari, Kelly Yap, Sumanta Kumar Pal

**Affiliations:** 1 Department of Medical Oncology & Experimental Therapeutics, City of Hope Comprehensive Cancer Center, Duarte, CA 91107, USA; nsalgia@coh.org; 2 University of California San Francisco School of Medicine, San Francisco, CA 94143, USA; errol.philip@ucsf.edu; 3 Department of Medical Oncology & Experimental Therapeutics, City of Hope Comprehensive Cancer Center, Corona, CA 92879, USA; mziari@coh.org; 4 Department of Medical Oncology & Experimental Therapeutics, City of Hope Comprehensive Cancer Center, South Pasadena, CA 91030, USA; keyap@coh.org

**Keywords:** renal cell carcinoma, team medicine, translational research, community practice, clinical trials

## Abstract

The treatment of metastatic renal cell carcinoma (mRCC) has rapidly evolved; however, the progress made in the field is heavily contingent upon timely and efficient accrual to clinical trials. While a substantial proportion of accrual occurs at tertiary care centers, community sites are playing an increasing role in patient recruitment. In this article, we discuss strategies to optimize collaborations between academic and community sites to facilitate clinical research. Further, as the role of biomarker discovery has become increasingly important in tailoring therapy, we will discuss opportunities to bridge diverse accrual sites for the purpose of translational research.

## 1. Introduction

Two decades ago, the outlook for patients with metastatic renal cell carcinoma (mRCC) was bleak. Treatments such as interferon-α (IFN-α) and interleukin-2 (IL-2) yielded a median survival of approximately one year [1]. The landscape changed tremendously with the introduction of targeted therapies from 2005 onwards, and, once again, with the introduction of checkpoint blockade a decade later [2,3,4,5]. In 2020, the current standard for front-line treatment of mRCC remains either dual checkpoint inhibition or a combination of targeted therapy with checkpoint blockade [6,7,8]. Survival estimates have essentially tripled what was anticipated in the cytokine era.

The rapid developments in mRCC therapy have been made possible by the timely and efficient completion of both early and late phase clinical trials. In addition, the discovery of novel therapies has been fostered by translational research efforts—the intersect of the bench and bedside. This is especially true in RCC, which has directly benefitted from research discoveries that have led to both the 2018 and 2019 Nobel Prizes in Physiology or Medicine. While tertiary centers (e.g., cancer centers and academic hospitals) serve as a common ground for early phase clinical trials and translational work, there is no doubt that their efforts can be bolstered through the participation of community oncology practices. Further, late-phase clinical trials, which often require less stringent data collection and less intensive evaluation schedules, can perhaps be done equally well at community and academic sites.

Whereas the prognosis of mRCC has improved, it is important to note that most patients with this disease are not cured, and thus, further research is imperative. In the current manuscript, we review the clinical landscape for renal cell carcinoma and delve into opportunities to maximize collaborations between community-based practices and academic sites in the hope of promoting more efficient and effective research endeavors. We approach the latter objective in the specific context of City of Hope—a tertiary cancer center that serves the greater Los Angeles area, a catchment that includes approximately 20,000,000 people. City of Hope has within its network 30 community-based satellite clinics, stretching over a radius of 100 miles [9]. With this vast network of community sites and the robust patient population that comes with it, multiple opportunities for collaborative research exist. However, selecting the right research for community versus academic practices is no small feat. Whereas our academic practice at City of Hope is well-staffed, with 5 research nurses and 7 clinical research associates devoted to genitourinary cancers, community sites often have a limited number of research staff who are responsible for managing studies across multiple disease types. These limitations notwithstanding, the integration of academic and community centers has grown into a successful model for conducting clinical and translational research.

## 2. Renal Cell Carcinoma Incidence and Histology

Renal cell carcinomas are a broad classification referring to tumors originating from the renal pelvis and medulla. The incidence of RCC is increasing, with an estimated 73,750 newly diagnosed cases in 2020 in the United States [10]. The disease now accounts for 5% of cancer diagnoses in men and 3% in women. The rising incidence of RCC can be partially attributed to an increase in the incidental detection of small renal masses during the performance of abdominal imaging for non-specific complaints [11]. Although the majority of incidental findings are small, early-stage masses, these diagnoses have not led to a substantial change in the mortality rate attributed to RCC; in 2020, it is estimated that RCC will account for approximately 15,000 deaths in the United States [10].

The histological variations in RCC have been well-documented [12]. Clear cell RCC (ccRCC) represents the most common subtype, accounting for 80% of diagnoses. ccRCC is pathologically characterized by its clear cell histology and acinar growth patterns. The hallmark molecular alterations of ccRCC include chromosome 3p loss and inactivation of the Von-Hippel Lindau (VHL) gene, which lies at the 3p25 locus [13]. VHL inactivation can occur either somatically or in the germline. Inactivation of *VHL* has been reported in up to 90% of sporadic ccRCC cases [14,15]. Germline inactivations of *VHL* are associated with Von-Hippel Lindau syndrome, which predisposes individuals to bilateral renal masses, as well as hemangioblastomas and retinal angiomas [16]. Other mutations associated with ccRCC are *PBRM1*, *SETD2*, and *BAP1*, all of which also lie on chromosome 3p [15,17].

Non-clear cell RCC (nccRCC) is a heterogenous classification that encompasses the other 20% of RCC diagnoses. Papillary RCC (pRCC) composes the majority of nccRCCs (~15% of total RCC diagnoses). pRCC has historically been subdivided into two classifications based on histology: type 1 involves low-grade nuclei arranged in a single layer, and type 2 encompasses a wide range of morphological presentations that can be further divided into at least three additional subtypes. Type 1 pRCC has been associated with sporadic mutations in the MET protooncogene (10–13% of patients) [18]. Other nccRCCs include chromophobe RCC, MiT translocation RCCs, collecting duct carcinoma, and renal medullary carcinoma. Each of the above-listed subtypes accounts for less than 10% of all RCC diagnoses [19].

As ccRCC represents the overwhelming majority of RCC diagnoses, the remainder of this review will focus primarily on the management of clear cell disease and use RCC interchangeably for ccRCC.

## 3. Managing Localized Renal Cell Carcinoma

Localized RCC comprises the majority of RCC cases, and in turn, is typically cured with definitive treatment. The standard of care for most localized tumors has been nephrectomy, whether it be partial or radical. Cancer-specific survival rates for localized RCC patients have been estimated to be 84% at five years and 76% at ten years after surgical resection, varying widely with stage. Although these estimates are overwhelmingly positive compared to patients with metastatic disease, one area of research interest has been the potential utility of adjuvant or neoadjuvant therapies to increase disease-free and overall survival (OS). A variety of agents, including IFN-α, IL-2, and chemotherapies, have been trialed as adjuvant therapies for localized RCC, but none demonstrated an improvement in disease-free survival. As the treatment landscape for mRCC has evolved, so too have the investigatory agents for adjuvant and neoadjuvant therapy.

The ASSURE trial and S-TRAC trial both compared adjuvant sunitinib to placebo in patients with high-risk localized RCC [20,21]. ASSURE also contained a third arm (sorafenib). No difference in disease-free survival (5.8 versus 6.1 versus 6.6 years for sunitinib, sorafenib, and placebo, respectively) nor OS was seen in ASSURE, and substantial toxicities were associated with both sunitinib and sorafenib. However, S-TRAC reported a statistically significant increase in disease-free survival with sunitinib versus placebo (6.8 versus 5.6 years) on independent review, but still no difference in OS. Based on the results of S-TRAC, sunitinib was approved for adjuvant use. Although approved, sunitinib is not often leveraged in the adjuvant setting due to the substantial toxicities associated with its use. Various other trials have investigated small molecule inhibitors as adjuvant therapy: PROTECT compared pazopanib to placebo, ATLAS compared axitinib to placebo, and EVEREST compared everolimus to placebo [22,23,24]. Both PROTECT and ATLAS have reported out and did not demonstrate improved survival with adjuvant therapy, while EVEREST has completed accrual, but results have not yet been reported.

**Figure 1:**
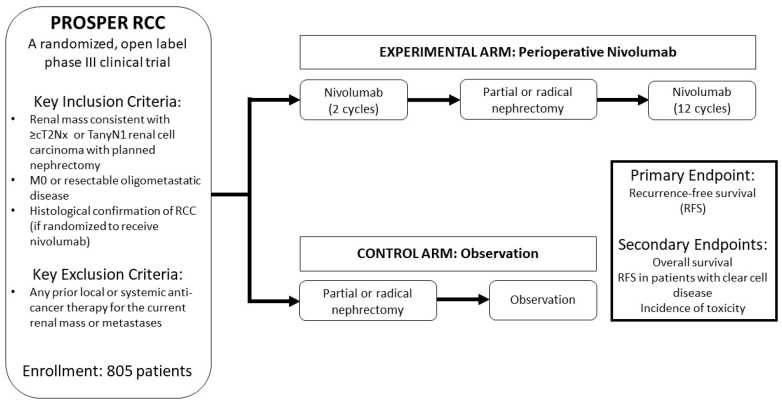
Schema for the phase-III ECOG-ACRIN PROSPER study.

Additional investigation of adjuvant therapy for localized disease has incorporated immune checkpoint inhibitors (CPIs). The recently opened PROSPER RCC trial randomizes patients to receive surgery alone or surgery with perioperative nivolumab (Figure 1) [25]. This study continues to accrue patients. IMmotion 010 also has investigated the role of adjuvant CPI therapy for resected RCC, in this case, comparing atezolizumab to placebo for patients with high-risk of disease recurrence [26]. This trial has not reported results to date. As the disclosed trials have reported minimal or no improvement with adjuvant therapy for localized RCC, systemic therapy is primarily isolated to patients with metastatic disease.

## 4. The Evolving Management of Metastatic Renal Cell Carcinoma

mRCC carries a relatively poor long-term survival (12% five-year OS) when compared to localized disease (93% five-year OS)—of course, these estimates are changing with emerging therapies. The systemic therapy landscape for mRCC has rapidly evolved within the last twenty years [27]. At the turn of the century, cytokine-based approaches with high-dose IL-2 and IFN-α represented the best systemic therapeutic option for patients with advanced disease. Both cytokines have been shown in animal and human models to have anti-cancer properties: IL-2 enhances the growth and activation of natural killer cells and CD-8+ T-cells, while IFN-α enhances tumor antigen presentation, among other characteristics [28,29]. While such cytokine approaches promote tumor regression mechanistically, IL-2 and IFN-α both provided limited clinical efficacy, with a median OS of approximately one year and durable responses in the range of only 5–7% [1,30,31]. These agents also posed substantial toxicity profiles, further limiting successful therapeutic outcomes.

### 4.1. Risk Stratification: MSKCC and IMDC Criteria

Among the most important non-therapeutic advances in the management of mRCC have been the development and implementation of risk classification algorithms. The Memorial Sloan Kettering Cancer Center (MSKCC) Score for renal cell carcinoma represents the first widely-adopted calculation for risk stratification of mRCC patients [31]. The initial MSKCC risk score included five prognostic factors: low Karnofsky performance status, high serum lactate dehydrogenase, low hemoglobin, high serum calcium, and absence of prior nephrectomy. These were used as risk factors to stratify patients into three groups: favorable risk (0 risk factors), intermediate risk (1–2 risk factors), and poor risk (3+ risk factors). Patients with MSKCC favorable risk disease were reported to have a median OS of 20 months, those with intermediate risk had a median OS of 10 months, and poor risk disease carried a median OS of 4 months. The MSKCC model has incorporated various additions into its algorithm over time. The Mekhail extension added two variables: prior radiotherapy and 2+ sites of metastasis [32]. In 2018, the MSKCC risk model was updated to incorporate genomic characteristics, specifically the mutational status of *BAP1*, *PBRM1*, and *TP53* in the stratification algorithm [33]. The MSKCC risk model has been used as an important inclusion criterion and stratification variable for patient randomization in various RCC therapeutic trials [5].

A second prognostic model has been developed and validated for mRCC: the International Metastatic Renal Cell Carcinoma Database Consortium (IMDC) Risk Score. Like the MSKCC model, the IMDC score utilizes clinical variables as prognostic markers to define patient risk. The IMDC model incorporates six prognostic variables: less than one year from the time of diagnosis to onset of systemic therapy, low Karnofsky performance status, low hemoglobin, high calcium, high neutrophil, and high platelet levels [34]. Similar to the MSKCC model, patients are classified into one of three risk groups. Favorable risk (0 factors) carries a median OS of 43.2 months, intermediate risk (1–2 factors) carries a median OS of 22.5 months, and poor risk (3+ factors) carries a median OS of 7.8 months [35]. Like the MSKCC model, the IMDC score has been adopted as a key inclusion criterion and stratification metric for many pivotal RCC trials [6].

### 4.2. VEGF Tyrosine Kinase Inhibitors, Multi-Kinase Inhibitors, and mTOR Inhibitors

The first shift in the therapeutic approach to mRCC occurred with the advent of small-molecule inhibitors that bind to and inhibit the activity of membranous receptors and intracellular proteins. The primary class of small molecule inhibitors in the mRCC treatment armamentarium are vascular endothelial growth factor–tyrosine kinase inhibitors (VEGF–TKIs). Mechanistically, *VHL* mutations lead to decreased ubiquitinylation of hypoxia-inducible factor (HIF) and upregulation of the circulating VEGF molecule which then binds the VEGF receptor, promoting angiogenesis [36,37]. VEGF–TKIs inhibit the tyrosine kinase domain of the VEGF receptor, and in turn, block the intracellular signaling cascade that promotes angiogenesis and cell division

Multiple VEGF–TKIs have been approved for mRCC. One benchmark phase-III study compared the VEGF–TKI sunitinib to IFN-α in patients with previously untreated mRCC [2]. Sunitinib was demonstrated to prolong progression-free survival (PFS) compared to IFN-α (11 versus 4 months) and was associated with a higher objective response rate (ORR) (31% vs. 6%). The results of this study led to the approval of sunitinib for first-line mRCC patients. Other VEGF–TKI agents approved in mRCC include sorafenib, pazopanib, and axitinib. While these agents have prolonged PFS and produced improved response rates compared to the previous standard-of-care cytokine therapies, VEGF–TKIs are not curative, and patients are susceptible to disease progression upon the development of drug resistance.

An additional class of targeted therapies, so-called multi-kinase inhibitors have also been approved in mRCC. These agents not only act as VEGF–TKIs but also inhibit the tyrosine kinase domains of additional protooncogenes [38]. Cabozantinib is a multi-kinase inhibitor with activity as a VEGF–TKI and also as an inhibitor of MET and AXL, both of which are associated with resistance to VEGF–TKIs. Cabozantinib was first approved for mRCC patients with treatment-refractory disease but was soon trialed as first-line therapy. The phase-II CABOSUN trial compared cabozantinib to sunitinib in the front-line setting [39]. This study met its primary endpoint of improvement in PFS with cabozantinib (8.6 versus 5.3 months) and demonstrated a higher ORR with cabozantinib based on an independent review (20% versus 9%). These results led to the approval of cabozantinib across all lines of therapy for patients with mRCC. However, like sunitinib and other VEGF–TKIs, cabozantinib has limited curative potential. As such, the approach of managing mRCC in the front-line with VEGF–TKIs and multi-kinase inhibitors has been replaced by the recent introduction of immune checkpoint inhibitors.

The mammalian target of rapamycin (mTOR) represents a highly-important intracellular target of mRCC therapy. mTOR is an enzymatic intermediate in the PI3K/AKT/mTOR signaling pathway that regulates the cell cycle [40]. Dysregulation of this pathway is a metabolic feature of many RCC tumors, making the components of its signaling cascade viable targets for pharmacologic inhibition. Two therapies approved for the management of mRCC act in this manner on mTOR: everolimus and temsirolimus. Everolimus is an oral agent that has been approved in combination for mRCC with the VEGF–TKI lenvatinib, following the results of a phase II study comparing the combination to each respective monotherapy [41]. Everolimus with lenvatinib resulted in PFS benefit compared to everolimus alone (14.6 versus 5.5 months) for patients who had received at least one prior VEGF–directed therapy. Temsirolimus, an inhibitory ester analog of mTOR that is applied intravenously, is also approved for mRCC [42]. A phase III trial demonstrated an OS benefit for temsirolimus over IFN-α (10.9 versus 7.3 months) with fewer incidences of serious adverse events [43]. Although no longer a mainstay of early-line therapy, the mTOR inhibitors everolimus and temsirolimus remain important interventions for the management of mRCC. Like VEGF–TKIs and multikinase inhibitors, however, mTOR inhibitors have given way to immune checkpoint inhibitors for the early-line treatment of mRCC.

### 4.3. Immune Checkpoint Inhibitors

Immune checkpoint inhibition is a relatively recent development in solid tumors, first explored in the context of melanoma [44]. CPIs are synthesized monoclonal antibodies capable of overcoming T-cell inactivation to elicit an anti-tumor response [45]. Approved CPIs exhibit activity on one of two immunoregulatory axes: programmed-death-1/programmed-death-ligand-1 (PD-1/PD-L1) and the cytotoxic-T-lymphocyte-associated protein 4 (CTLA-4). The treatment paradigm for mRCC has advanced greatly since the advent of PD-(L)1 and CTLA-4 inhibitors.

CheckMate-025 was the first study to investigate nivolumab, an anti-PD-1 CPI, in mRCC [5]. This study enrolled patients who had progressed on one or two previous VEGF–TKIs and randomized them to receive nivolumab or everolimus. Nivolumab outperformed everolimus in OS (25 versus 19.6 months) and ORR (25% versus 5%), leading to the approval of nivolumab monotherapy for mRCC patients who had progressed on previous therapy.

The ensuing CheckMate-214 trial compared the combined regimen of nivolumab with ipilimumab, an anti-CTLA-4 CPI, to sunitinib in previously-untreated mRCC [6]. Patients were stratified based on the International Metastatic Renal Cell Carcinoma Database Consortium (IMDC) risk score. In patients with intermediate or poor risk disease, nivolumab + ipilimumab greatly outperformed sunitinib, demonstrating greater ORR (42% versus 27%) and a rate of complete response totaling 9%. However, among patients with favorable risk disease by IMDC criteria, sunitinib was shown to result in prolonged PFS (25.1 versus 15.3 months) and a greater ORR (52% versus 29%). The results from CheckMate-214 established a new treatment algorithm for front-line mRCC, in which dual checkpoint inhibition with nivolumab + ipilimumab form the backbone of therapy for intermediate/poor risk patients but VEGF–TKIs remain a viable first-line option for patients with favorable risk.

### 4.4. Combination Therapies

Further endeavors have clinically investigated combinations of CPIs with VEGF–TKIs. A myriad of large phase III clinical trials have been undertaken, with three having reported out in early 2019. The IMmotion 151 trial compared the investigatory combination of atezolizumab (an anti PD-L1 CPI) with bevacizumab (an anti-VEGF monoclonal antibody) against sunitinib [46]. This trial met its primary endpoint of improvement in PFS with bevacizumab + atezolizumab (11.2 versus 7.7 months) in the PD-L1+ population. In the intention-to-treat cohort that included all patients regardless of PD-L1 status, PFS favored the combination therapy over sunitinib (11.2 versus 8.4 months). JAVELIN-101 followed a similar design model, comparing the combination of avelumab (an anti-PD-L1 CPI) and axitinib against sunitinib in patients with mRCC who had received no prior therapy [8]. The primary endpoint of improved PFS with axitinib + avelumab in patients who had PD-L1+ disease was met (13.8 versus 7.2 months). Likewise, irrespective of PD-L1 status, axitinib + avelumab was again associated with improved PFS (13.8 versus 8.4 months).

The KEYNOTE-426 trial utilized an experimental arm of pembrolizumab (an anti-PD-1 CPI) with axitinib, which was compared against sunitinib [7]. The axitinib + pembrolizumab combination resulted in a prolonged PFS compared to sunitinib (15.1 versus 11.1 months), and, additionally, ORR was greater in the axitinib + pembrolizumab arm (59% versus 36%). These three trials all reported within the span of two months in 2019. Table 1 summarizes results from trials testing CPIs (in combination with an additional CPI or with VEGF–TKIs) for the first-line treatment of mRCC. A recent press release in April 2020 has also indicated that the CheckMate-9ER trial (NCT03141177) of nivolumab + cabozantinib versus sunitinib for front-line mRCC has met its primary endpoint of improved PFS and secondary endpoints of ORR and OS. The combination of axitinib + pembrolizumab, in particular, has already established a role in the mRCC treatment algorithm for front-line intervention in patients with IMDC good-risk disease [47]. As data from these trials continue to mature and additional trials are undertaken, it is reasonable to believe the approval and usage of combination therapies will expand.

### 4.5. Cytoreductive Nephrectomy for Metastatic Disease: A Continuing Discussion

The utility of cytoreductive nephrectomy as a component of care for mRCC remains a highly-investigated topic. Cytoreductive nephrectomy for removal of the primary tumor in patients with metastatic disease was a standard-of-care for twenty years. However, two recent trials, SURTIME and CARMENA, have changed the paradigm for nephrectomy in mRCC. SURTIME compared immediate nephrectomy (followed by sunitinib therapy) versus deferred nephrectomy (preceded by sunitinib therapy) [48]. The results of this study indicated that deferred nephrectomy did not improve the progression-free rate at 28 weeks compared to immediate nephrectomy (43% versus 42%), but OS was higher in the deferred arm (32.4 versus 15.0 months). These results suggested that initial systemic therapy followed by a potential debulking nephrectomy may be more efficacious for mRCC patients than the former standard of upfront nephrectomy.

CARMENA randomized patients with mRCC to undergo nephrectomy followed by sunitinib or to receive sunitinib alone [49]. Results from this study indicated that sunitinib alone offered a non-inferior alternative to nephrectomy followed by sunitinib, with OS favoring the former (18.4 versus 13.9 months). The results of CARMENA and SURTIME have led to a shift away from cytoreductive nephrectomy as a standard-of-care for mRCC, except in carefully planned cases. However, ongoing trials continue to study the relevance of cytoreductive nephrectomy. The NORDIC-SUN trial (NCT03977571) investigates nephrectomy following therapy with nivolumab + ipilimumab. Patients with IMDC favorable- or intermediate-risk disease are randomized to receive nephrectomy or maintenance nivolumab. Patients who present with poor-risk disease can be randomized within this study as well, so long as their risk classification is adjusted to intermediate-risk following initial therapy with nivolumab + ipilimumab or while on maintenance nivolumab. An additional trial, CYTOSHRINK (NCT04090710), uses stereotactic body radiation therapy (SBRT) as an investigational definitive treatment of the primary tumor site for patients with mRCC. In this trial, patients are randomized to receive the standard-of-care nivolumab + ipilimumab for four cycles, followed by maintenance nivolumab or the experimental arm. The experimental arm consists of one cycle of nivolumab + ipilimumab followed by five fractions of SBRT to the kidney lesion before resuming nivolumab + ipilimumab on the standard schedule. Both these trials are currently open to accrual.

## 5. Integrating Community and Academic Practices for Renal Cell Carcinoma Research

As RCC incidence continues to grow, the role of the community oncologist has grown increasingly important in the network of care for RCC patients. While clinical and translational research has primarily been led by academic oncologists, a new approach incorporating community practitioners into the research paradigm is now viable. It is important that medical oncologists at academic centers integrate community practitioners, particularly urologists and oncologists, into a collaborative model that promotes exceptional, multi-disciplinary care alongside cutting-edge clinical and translational research. Through the City of Hope network, which encompasses a central academic center and a diffuse network of community sites, we have developed a collaborative model for conducting translational research and clinical trial accrual that integrates academic and community oncologists. Below, we have highlighted our experiences utilizing this integrated structure to advance clinical and translational research and discovery in RCC.

## 6. Collaborations in Translational Research

As detailed above, the therapies currently in use for mRCC reflect two biological principles, namely, that (1) RCC is driven by angiogenesis and that (2) defects in the immune system can propagate the disease. One of the genomic hallmarks of RCC are defects in the *VHL* gene [15,50]. Mutation of *VHL* (largely sporadic, but possibly hereditary) leads to decreased ubiquitinylation of HIF [51,52,53,54]. This, in turn, leads to the upregulation of vascular endothelial growth factor (VEGF), a potent mediator of angiogenesis [55,56]. The interplay of RCC with the immune system is much more complex. Multiple studies have shown varying levels of expression of the immune checkpoint programmed death-ligand 1 (PD-L1) in RCC tissues [7,57,58]. Interaction of PD-L1 with its cognate receptor, programmed death-1 (PD-1), leads to T-cell anergy, which can be overcome with antibodies targeting either entity [59,60,61].

**Figure 2:**
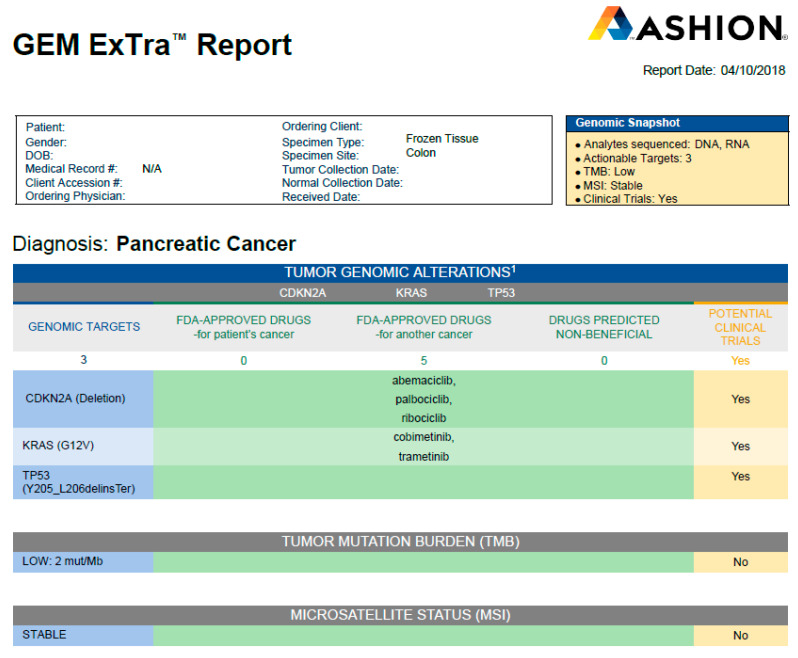
A sample report from the Ashion GEM ExTra test.

Interestingly, while VEGF–pathway inhibitors and immune checkpoint inhibitors both reflect “targeted therapies”, both are applied in a biomarker-agnostic fashion in patients with mRCC. At City of Hope, a partnership has been forged with TGen, Inc., a company focused on translational genomics. Scientists and clinicians at TGen have developed the Ashion GEM ExTra^®^ platform that allows for both whole-exome sequencing and RNA sequencing [62]. A sample report is shown in Figure 2. In addition to requiring tissue for tumor sequencing, the platform uses blood for germline correction.

A sequencing platform such as Ashion GEM ExTra^®^ offers two benefits in terms of translational research. First and foremost, collecting this data and pooling it across academic and community sites may allow for the development of predictive and prognostic biomarkers. In a recent effort, Salgia et al. reviewed data from 90 patients with mRCC (Salgia et al. ASCO 2020) and examined the association between treatment type and response among those that had received immunotherapy or targeted therapy. Notably, DNA level data suggested that *TERT* promoter alterations were a negative predictor of immunotherapy response, with RNA level data from the study identifying multiple *TERT*-associated pathways that could play a role in immunotherapy resistance.

A further benefit of amassing translational data across community and academic sites is the ability to identify novel opportunities for clinical research. Our pooled data indicate, for instance, a preponderance of mutations in *VHL*, *PBRM1*, *SETD2*, and other mutations in the mammalian target of rapamycin (mTOR) pathway. We have also observed infrequent but clinically significant mutations in genes such as *EGFR*, *MET*, and *ALK* [63,64]. Many phase I and II trials in development focus specifically on these mutations but do so in a tumor agnostic fashion. The genitourinary group physicians at our institution have partnered with community sites and other disease teams to categorize cumulative mutational frequency. By pooling this data across our sites, we can make logical decisions regarding the most appropriate clinical trials to bring into our portfolio.

Biomarker discovery and validation are not isolated to genomics, however. Recent work has elucidated associations between microbiome composition and clinical response to CPIs in mRCC [65]. Additional work from our group and collaborators at TGen, Inc. has investigated the enteric microbiome as a potential biomarker for responses to the above-mentioned therapy regimens in RCC [66]. We have utilized whole genome shotgun metagenomic sequencing to correlate the stool microbiome profile and microbiome diversity of mRCC patients with clinical benefit, both for patients receiving VEGF–TKIs and for patients receiving CPIs (Dizman et al. ASCO 2020). In these studies, we have identified a variety of microbial species (such as *B. Intestihominis* and *B. adolescentis*) that are correlated with clinical benefit in patients receiving systemic therapy for mRCC. The diversification of biomarker pursuits provides another avenue for research collaboration between academic and community partners. Protocols for the collection, storage, and shipment of stool, for example, can be implemented at any site, even those with limited research infrastructure. The clinical volume of community practice sites poses an untapped resource for furthering biomarker discovery and other translational studies in RCC.

## 7. Collaborations in Clinical Research

### 7.1. Phase I and II Clinical Trials

**Figure 3:**
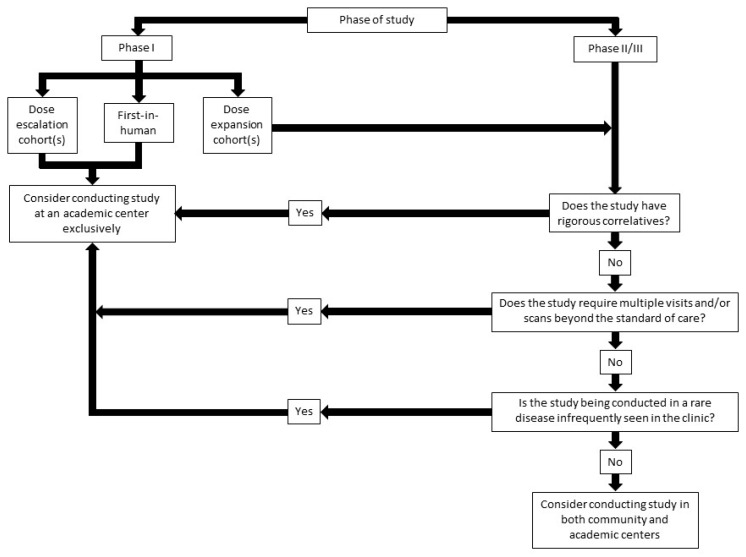
Schema outlining appropriate trial selection for community and academic settings.

Phase I clinical trials have morphed in recent years, moving away from the classical 3 + 3 design [67]. More recent iterations of phase I clinical trials have started with a dose-escalation phase, expanding quickly into phase Ib trial designs, all while seeking validation in disease- or mutation-specific settings. We propose that phase I studies in dose escalation are perhaps most appropriate to remain at academic centers. The dose-escalation phase is one where rigorous oversight is necessary, with multiple visits and correlative studies. Furthermore, this is often the phase in which novel toxicities associated with an agent or combination are recognized and documented. We propose that highly selected phase Ib studies could be conducted in the community (Figure 3). Criteria for site selection in these studies must account for the frequency of study visits and the rigor of correlatives. For instance, if a proposed study requires pharmacokinetic and pharmacodynamic blood assessments on hourly intervals (not unusual in a phase-I protocol), the trial may be challenging in a busy community practice. Similarly, if a study requires repeat biopsies or other invasive assessments, academic centers may be better suited.

Indeed, there are real-world examples of phase I studies that may be too challenging to envision in a community practice setting at this time. For instance, our genitourinary cancers group is heavily invested in studies of chimeric antigen receptor (CAR)–T-cell therapies (Dorff et al. ASCO GU 2020). In addition to the complexities associated with the manufacturing and storage of CAR–T-cells, patients must be kept in an inpatient setting for prompt recognition and treatment of unique side effects of this treatment, such as cytokine release syndrome [68]. At present, commercially available CAR–T-cell therapies are used in a limited subset of patients with hematologic malignancies [69]. Until these therapies are adopted in a much more widespread fashion in the community (with appropriate infrastructure and monitoring), we recommend these studies remain within academic centers.

The phase II study is a vanishing entity, as companies often now move quickly from a large phase Ib effort to phase III. This is true in mRCC space as well—as one example, COSMIC-021 (NCT03170960) is an international study chaired by investigators at our site. This phase Ib study of cabozantinib with atezolizumab enrolled cohorts in RCC, lung cancer, and prostate cancer, as well as more than a dozen other histologies. Based on significant activity in the three noted cohorts, phase III clinical trials have been rapidly launched, including CONTACT-03 (NCT04338269) for mRCC [70]. In general, if phase II studies are to be considered in the community setting, the same factors (visit frequency, correlative studies, and so on) should aid in deciding whether studies are appropriate and should proceed.

### 7.2. Phase III Clinical Trials

Phase III clinical trials represent an area where collaborations are imperative. These studies often include hundreds (if not thousands) of patients, and to accrue in a rapid fashion, a partnership between academic and community sites is essential. There are two general categories of phase III studies that we consider in our program—one falls under the umbrella of pharmaceutical research, and the other relies upon funding from the National Cancer Institute (NCI), typically through the cooperative group mechanism. The cooperative groups have recently been consolidated—several groups (SWOG, Alliance, NRG, ECOG-ACRIN, and COG) retain members at both academic and community sites.

In general, cooperative group studies have more recently adopted easier thresholds for enrollment and less intensive patient follow-up. An example of this is the phase III PROSPER study, run by ECOG-ACRIN, which we have previously highlighted [25]. This phase III trial compares surgery alone for localized or oligometastatic RCC to surgery with both preoperative and postoperative nivolumab, a PD-1 inhibitor (Figure 1). The study has relaxed eligibility criteria with recent amendments—with randomization possible prior to a histologic diagnosis, and a pretreatment biopsy only required if patients are randomized to perioperative therapy. The study also does not mandate central review.

Such eligibility requirements differ widely from comparable pharmaceutical trials. For example, investigators at our institution led the phase III Immotion010 clinical trial, a study comparing atezolizumab to placebo in patients with completely resected RCC [26]. The study mandated pre-registration assessment of PD-L1 status, and further required central submission of scans. Not only do such measures increase the potential rate of screen failures, but they also increase the complexity of trial administration. Submitting tissue and scans in the pre-registration period (typically 2–3 weeks) is a wieldy task, and community sites must establish whether they have adequate resources to devote to such tasks.

Further, factors such as the study population, in particular tumor histology, must also be carefully considered in the conduct of trials. For example, studies investigating rare tumor histologies (nccRCCs) are challenging to conduct in the community setting due to the infrequency of disease presentation. One such example is SWOG 1500 (NCT02761057), a study of selective MET-kinase inhibitors for patients with papillary renal cell carcinoma that was chaired by physicians in our genitourinary oncology disease team. Although the study was sponsored by an NCI cooperative group, the relative rarity of papillary histology (~10–15% of metastatic renal cell carcinomas) dictated that the study only be available at the academic center [71]. As patients with rare histologies are often referred from the community to experts at academic centers, consolidation of trial sites in these instances should be considered.

## 8. Optimizing Partnerships between Community and Academic Sites

As noted previously, the academic cancer center is typically well staffed with multiple research nurses and clinical research associates who work within a single disease space. Each has ownership over a selected number of protocols (typically 2–3), and there are often regular meetings with the principal investigator. These face to face meetings with principal investigators offer an opportunity for education regarding the protocol and, furthermore, allow for a closer degree of oversight of protocol-related activities. At City of Hope, investigators in the genitourinary disease team frequently host didactic sessions for research staff, offering them insight into the current standard management of RCC, bladder, and prostate cancer. As research staff are often charged with screening for enrollment or suggesting toxicity attributions, understanding the underlying disease and associated treatment landscape is critically important.

Models of research in community practices vary but commonly require research staff to manage a much broader swath of clinical protocols across multiple cancer types. Furthermore, the principal investigator for a study may not reside at the community practice site, and thus is unable to engage with clinical and administrative staff on a routine basis. At City of Hope, the vast majority of principal investigators reside at the main campus; thus, elements of study oversight (filing significant adverse events, reporting study deviations) may be somewhat delayed at community sites. In addition, services readily available at the main campus may be less accessible in the community practices; for example, labs may be obtained off-site through third-party vendors. This can create delays in lab reporting, and complicate study protocols that require same-day lab results to issue therapy. Many protocols also embed correlates that require additional processing methods (e.g., centrifugation for separation of plasma aliquots).

Similarly, access to appropriate and timely scans can vary by recruitment location, with an academic center generally able to accommodate access to both standard and novel imaging tests (e.g., fluciclovine or PSMA PET scans for prostate cancer) and not have to rely on third parties to transfer images so that relevant tumor measurements can be obtained. Whereas these may be trivial issues on a main academic campus, access to a centrifuge, processing equipment, and appropriate imaging modalities may be limited at community sites.

Despite these noted limitations, community practices can serve as valuable contributors to clinical research endeavors housed at a main campus. To optimize this relationship and promote successful collaborations, we have implemented several strategies. Firstly, self-nominated community disease team leads are identified to serve as liaisons between the research team on the main campus and oncologists practicing in the community. These individuals also play a role in determining the feasibility of specific clinical trials in the community, taking into account many of the considerations that we have outlined throughout. In addition, these individuals are invited to bimonthly genitourinary disease team meetings, where the group conducts a thorough review of all existing protocols and evaluates new clinical trial proposals. Finally, in recognition of the limited time permitted to community oncologists to learn about existing research endeavors, our genitourinary cancers group has established a newsletter that highlights ongoing and planned clinical trials with only the most salient information—basic eligibility requirements and study schema, for instance. These newsletters have triggered a number of direct referrals for clinical trial enrollment.

One relatively recent development among our community-based satellite clinics is the implementation of a standardized electronic health record (EHR) mirroring that at the main campus, optimizing the process by which physicians can evaluate and triage patients who may be suitable clinical trial candidates.

## 9. Conclusions

The clinical approach to RCC has drastically changed over the past two decades. The novel therapeutic options introduced to the medical oncologist’s armamentarium have leveraged ground-breaking work on angiogenesis and induced hypoxia in addition to the principle of immune checkpoint blockade to overcome T-cell anergy. To maintain the momentum of RCC drug development, rigorous translational research must continue advancing our understanding of RCC biology and clinical trials must continue to accrue patients at a rapid pace. As we have highlighted herein, this can happen effectively and efficiently if community practices are integrated with academic centers in the conduct of translational research and clinical trials. We have proposed a number of strategies to optimize this relationship, with the common theme being enhanced communication. When oncologists at community and academic practices frequently dialogue, gaining a deeper understanding of each other’s practice patterns, a portfolio of trials can be developed that optimizes accrual and advances scientific findings more rapidly.

The positive effects of an academic and community collaboration on trial accrual have been evidenced in our network—with our genitourinary cancers group recognized as the top accrual site for the adjuvant EVEREST study, a trial comparing everolimus and placebo in patients with resected localized RCC [24]. Our enrollment was driven in large part by patients seen at our community-based satellite offices. The rapid success of this collaborative effort was used as a foundation for the enrollment of patients into further adjuvant trials (e.g., E2810 and Immotion010), with similar positive outcomes. In sum, a strong collaborative approach between our academic center and affiliated community practices has resulted in a model conducive to high accrual rates across a broad range of clinical trials. This feat, along with the opportunities for collaboration in the translational sciences, underscores the benefit of these partnerships.

## Figures and Tables

**Table 1:** Results from four phase-III clinical trials investigating front-line immune checkpoint inhibitor use in metastatic renal cell carcinoma.

Trial	CheckMate-214 (Intermediate + Poor Risk Patients)		IMmotion-151		JAVELIN-101		KEYNOTE-426	
Arms	Nivolumab + Ipilimumab	Sunitinib	Bevacizumab + Atezolizumab	Sunitinib	Axitinib + Avelumab	Sunitinib	Axitinib + Pembrolizumab	Sunitinib
Patients Enrolled	425	422	454	461	442	444	432	429
ORR, % (95% CI)	42 (37–47)	27 (22–31)	37 (32–41)	33 (29–38)	51 (47–56)	26 (22–30)	59 (55–64)	36 (31–40)
Median PFS	11.6	8.4	11.2	8.4	13.8	8.4	15.1	11.1
PFS HR (95% CI)	0.82 (0.64–1.05) *		0.83 (0.70–0.97)		0.69 (0.56–0.84)		0.69 (0.57–0.84)	
OS HR (95% CI)	0.63 (0.44–0.89) ^†^		0.93 (0.76–1.14)		Not Reported		0.53 (0.38–0.74)	
Grade 3/4 Adverse Events, %	46	63	40	54	55	55	63	58

Abbreviations: ORR = overall response rate, CI = confidence interval, PFS = progression-free survival, HR = hazard ratio, OS = overall survival. * 99.1% confidence interval; ^†^ 99.8% confidence interval.
